# Multiple sulfur isotope evidence for massive oceanic sulfate depletion in the aftermath of Snowball Earth

**DOI:** 10.1038/ncomms12192

**Published:** 2016-07-22

**Authors:** Pierre Sansjofre, Pierre Cartigny, Ricardo I. F. Trindade, Afonso C. R. Nogueira, Pierre Agrinier, Magali Ader

**Affiliations:** 1 Équipe de géochimie des isotopes stables, Institut de Physique du Globe de Paris, Sorbonne Paris Cité, Univ Paris Diderot, UMR 7154 CNRS, F-75005 Paris, France; 2 Departamento de Geofísica, Instituto de Astronomia, Geofísica e Ciências Atmosféricas, Universidade de São Paulo, Rua do Matão 1226, São Paulo 05508-900, Brazil; 3 IUEM, Laboratoire Domaines Océaniques, Université de Bretagne Occidentale, 29280 Plouzane, France; 4 Faculdade de Geologia, Instituto de Geociências, Universidade Federal do Pará, CEP, Belém 66.075-110, Brazil

## Abstract

The terminal Neoproterozoic Era (850–542 Ma) is characterized by the most pronounced positive sulfur isotope (^34^S/^32^S) excursions in Earth's history, with strong variability and maximum values averaging δ^34^S∼+38‰. These excursions have been mostly interpreted in the framework of steady-state models, in which ocean sulfate concentrations do not fluctuate (that is, sulfate input equals sulfate output). Such models imply a large pyrite burial increase together with a dramatic fluctuation in the isotope composition of marine sulfate inputs, and/or a change in microbial sulfur metabolisms. Here, using multiple sulfur isotopes (^33^S/^32^S, ^34^S/^32^S and ^36^S/^32^S ratios) of carbonate-associated sulfate, we demonstrate that the steady-state assumption does not hold in the aftermath of the Marinoan Snowball Earth glaciation. The data attest instead to the most impressive event of oceanic sulfate drawdown in Earth's history, driven by an increased pyrite burial, which may have contributed to the Neoproterozoic oxygenation of the oceans and atmosphere.

## Abstract (web summary)

Large positive sulphur isotope excursions, recorded in the wake of the Marinoan glaciation have previously been interpreted assuming stable ocean sulphate concentrations. Here, using multiple sulphur isotopes, the authors instead suggest significant ocean sulphate drawdown, driven by increased pyrite burial.

## Graphical abstract

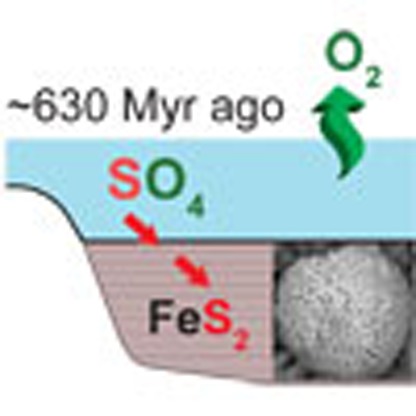

Sulfate represents one of the most important metabolic electron acceptors on the planet, and constraining its biogeochemical cycle is crucial for understanding the long-term redox evolution of the oceans and atmosphere^1^,^2^,^3^,^4^. Classically, the sedimentary record of sulfur cycling is probed through the sulfur isotopic composition of sedimentary pyrite, δ^34^S_pyr_, and carbonate-associated sulfate (CAS), δ^34^S_CAS_ (where δ^34^S=10^3^ × (^34^S/^32^S_sample_/^34^S/^32^S_Canyon Diablo Troilite_−1), both of which may record fluctuations in past marine sulfate isotope composition^5^. Variations in oceanic sulfate isotope composition (δ^34^S_SO4_) are usually interpreted under steady-state assumptions^6^,^7^,^8^,^9^,^10^, whereby the oceanic sulfate content (M_SO4_), here written for ^32^S, remains constant:


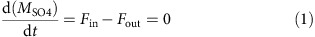


*F*_in_ being the input flux related to weathering and *F*_out_ the output flux related to pyrite and evaporite burial. Long-term variations in δ^34^S_SO4_ therefore depend on the fraction of sulfur buried as pyrite relative to evaporite (*f*_pyr,_ ranging from 0 to 1) as well as on the isotopic composition of sulfate delivered to the ocean (δ^34^S_in_), according to the conservative isotopic mass equation:


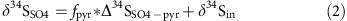


where Δ^34^S_SO4-pyr_=δ^34^S_SO4_−δ^34^S_pyr_ is the difference between the average δ^34^S_SO4_ of evaporites and/or CAS and the average δ^34^S_pyr_ of sedimentary pyrite at a given time. Considering a modern δ^34^S_in_ of +3‰, a Δ^34^S_SO4-pyr_ of 40‰, and a modern seawater δ^34^S_SO4_ of +21‰, the resulting present-day *f*_pyr_ is close to 0.45 (ref. 11).

In a steady-state framework (equation (2)) and assuming modern δ^34^S_in_ and Δ^34^S_SO4-pyr_ values, the strong positive δ^34^S_CAS_ recorded in Neoproterozoic^6^,^7^,^9^,^10^,^12^,^13^, sediments would only hold for *f*_pyr_ values above unity which are therefore inconsistent. A concomitant increase of both *f*_pyr_ and Δ^34^S_SO4-pyr_ for such excursions is thus necessary. Similarly, assuming that Δ^34^S_SO4-pyr_ is constant, the high δ^34^S_CAS_ values (accompanied by high δ^34^S_pyr_) need to be accounted by a concomitant increase in both *f*_pyr_ and δ^34^S_in_. For example, the δ^34^S_CAS_ positive Ara anomaly (∼545 Ma)^10^ in Oman requires anomalously high *f*_pyr_ (0.9) and δ^34^S_in_ (15‰) values for which a geological driver remains to be identified. Others have instead observed an increase in δ^34^S_CAS_ values not accompanied by a change in δ^34^S_pyr_ values, resulting in a Δ^34^S_SO4-pyr_ increase that may reflect the advent of sulfur disproportionation (SD) metabolism^7^,^8^. An alternative possibility is that the steady-state model itself does not hold for the Neoproterozoic. This hypothesis has already been envisaged^13^,^14^ but has never really been tested so far because it increases further the number of unconstrained parameters compared with the steady-state model (for example, δ^34^S_in_, Δ^34^S_SO4-pyr_, *F*_in_, *F*_out_ and M_SO4_).

Here we use multiple sulfur isotopes (^33^S/^32^S, ^34^S/^32^S and ^36^S/^32^S ratios) of CAS and pyrite to investigate dynamic models for the Neoproterozoic sulfate reservoir evolution in the aftermath of the Marinoan glaciation. The results show that the steady-state assumption does not hold in the aftermath of the Marinoan Snowball Earth glaciation and attest to an impressive event of oceanic sulfate drawdown.

## Results

### Stratigraphy and age constraints

The studied sedimentary sequence corresponds to the Mirassol d'Oeste and Guia formations (Araras group, Central Brazil) and starts with a typical cap dolostone (∼635 Ma, refs 15, 16, 17) directly overlying Marinoan glacial deposits. We focus primarily on CAS as its isotopic composition is generally considered to reflect a seawater signal^5^, provided that diagenesis and recrystallization are limited. Samples were selected based on previous petrographic and geochemical results^17^ to avoid as much as possible diagenetic overprints. Selected samples were then carefully washed to avoid contamination (Methods section). It has been shown that secondary pyrite oxidation to sulfate and atmospheric sulfur contamination may lead to lower δ^34^S_CAS_ values^18^,^19^. The consistent isotopic pattern displayed by the generated data set (see below) suggests that contamination was not significant in our samples.

**Figure 1:**
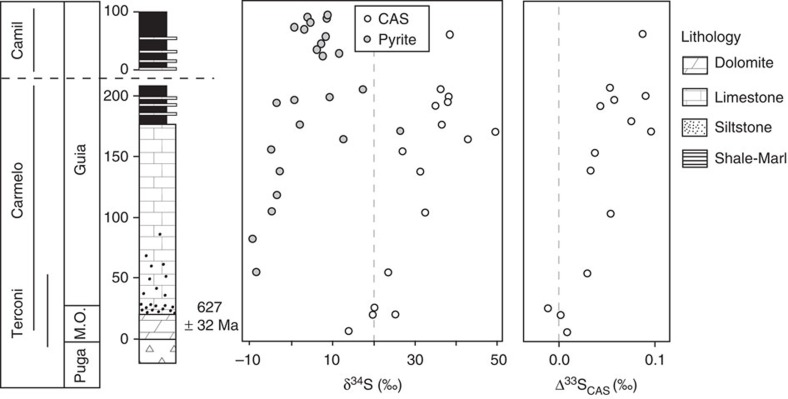
Isotopic results of sedimentary carbonates of the Araras platform. Sulfur isotope composition of sulfate (δ^34^S_CAS_ and Δ^33^S_CAS_) and pyrite (δ^34^S_pyr_) of the Araras carbonates. Results are given in ‰ versus CTD. Terconi, Carmelo and Camil section are represented as a single composite stratigraphic log^17^.

Three slightly overlapping sections were sampled in freshly exposed quarries (Terconi, Camil and Carmelo quarries) along a basinward profile, from the innershelf to the outershelf of the Araras carbonate platform^17^. The base of the composite section (Fig. 1) corresponds to the post-Marinoan glaciation diamictite-dolostone contact that is globally dated at 635.5 Ma (refs 20, 21). This is further constrained by Sr and C isotopes correlations and a Pb–Pb age of 627±32 Myr (see Supplementary Information of ref. 22). The age of the top of the section is constrained by the acritarch assemblage of the overlying Nobres Formation *(Cavaspina acuminata*, *Chlorogloeaopsis* sp., *Obruchevella* sp., *Ericiasphaera* sp., *Appendisphaera barbata*, *Tanarium irregulare, Tanarium conoideum* and *Micrhystridium pisinnum*^23^), which corresponds to the ECAP biozone of Grey (2005), ref. 24, that is, to an age interval of 570–580 Myr. This estimate is coherent with recently obtained detrital zircon and mica ages at 544±7 Myr on the overlying Diamantino Formation^25^. We can therefore reasonably estimate a maximum depositional time (from 635 to 575 Ma) of 60 Myr for Mirassol do Oeste and Guia formations (300 m of sediments). Outcrop-based facies analysis, complemented by petrographic description of representative samples, reveals a transgressive systems tract, with the deepest part of the platform corresponding to a ramp depositional environment^17^.

### Sulfur isotopes

We extracted sulfate from 16 micritic carbonate samples. Most CAS analyses (12 out of 16) were performed on samples from the Carmelo quarry, which contains the thickest post-glacial sedimentary sequence (Fig. 1). At the base of the section (that is, in the direct aftermath of glaciation), δ^34^S_CAS_ is +14‰ (lower than that of the modern ocean, ∼+21‰) and increases steadily up to ∼+38‰, locally reaching +50.6‰ (*n*=2, Fig. 1 and Supplementary Table 1). Our range of values is consistent with data reported for other Marinoan post-glaciation deposits in Namibia^6^ and North China^26^.

The isotope composition of pyrite was also analysed (*n*=35, from Terconi, Carmelo and Camil sections). Pyrite is absent from the first 50 metres of the composite section. Above the base, its content varies between 0.01% and 3.06%. Scanning electron microscopy investigations show that pyrite is present as a mixture of framboidal aggregates (that is, early diagenetic) and euhedral crystals^17^ (that is, later-diagenetic). δ^34^S_pyr_ increases upsection from −9.9 to +26.2‰, with strong variations in the upper part of the section that are associated with lithological variations (Fig. 1). δ^34^S_pyr_ and δ^34^S_CAS_ are broadly correlated (*R*^2^=0.79), yielding a mean Δ^34^S_CAS-pyr_ of 33.5±5.3‰ (1*σ*).

### Multiple sulfur isotopes

**Figure 2:**
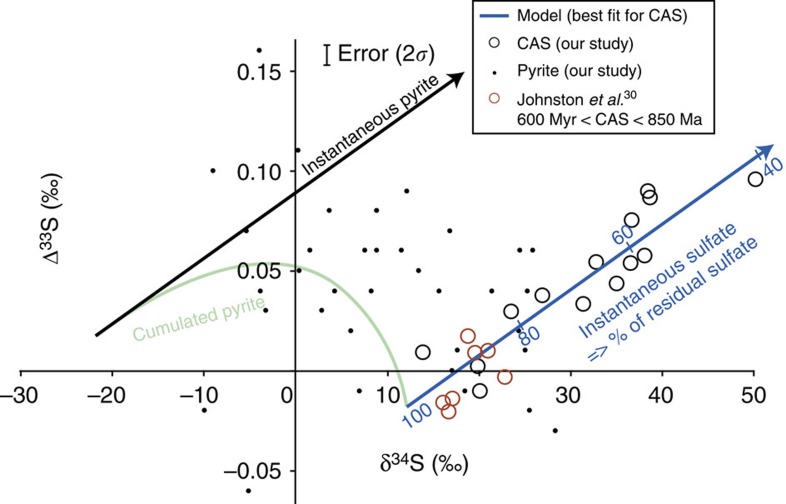
Multi-isotopic cross-plot and model results. Δ^33^S versus δ^34^S diagrams, best-fit line for CAS is obtained using the following parameters for the model: ^34^*α*_sulfide-sulfate_=0.960, ^33^*β*=0.5125 and *F*_in_/*F*_out_=0.3. The blue line corresponds to the evolution of the isotopic composition of the residual sulfate during a distillation. The black and green lines correspond to the isotopic evolution of the instantaneous and cumulative pyrite, respectively.

For the multiple sulfur isotopes analyses, Δ^33^S_CAS_ (where Δ^33^S=δ^33^S−10^3^ × ((δ^34^S/1,000+1)^0.515^−1), see ref. 27)) increases from −0.01‰ at the base to +0.10‰ at the top of the section (both±0.01‰, 2*σ*; Fig. 1). A clear positive correlation is observed between Δ^33^S_CAS_ and δ^34^S_CAS_ with *R*^2^=0.82 (Fig. 2). Δ^36^S_CAS_ (where Δ^36^S=δ^36^S−10^3^ × ((δ^34^S/1,000+1)^1.89^−1) varies between +0.09 and −0.46‰ (±0.1‰, 2*σ*) and correlates negatively with both δ^34^S_CAS_ and Δ^33^S_CAS_.

## Discussion

Deviations of Δ^33^S_CAS_ and Δ^36^S_CAS_ from zero together with correlations of δ^34^S_CAS_−Δ^33^S_CAS_ (Fig. 2), δ^34^S_CAS_−Δ^36^S_CAS_ and Δ^36^S_CAS_−Δ^33^S_CAS_ are observed for the first time in Neoproterozoic sections and result from mass-conservation effects^27^. Our results can only be produced in a non-steady-state system where *F*_in_≠*F*_out_, the non-zero Δ^33^S_CAS_ values being a consequence of the subtle interplay of sulfate input and removal from the ocean (Methods section). Sulfate input lowers the oceanic sulfate Δ^33^S_CAS_ through mixing processes (Supplementary Fig. 1), whereas sulfate removal from the ocean by hydrothermal or biological activity, the latter including bacterial sulfate-reduction (BSR) coupled to pyrite burial, increases the Δ^33^S_CAS_ of the residual oceanic sulfate (Supplementary Figs 2 and 3, ref. 27).

The analytical results and observed correlations can be quantitatively modelled by combining the dynamic equations of mass balance (equation (3)) and isotopic mass balance (for example, equation (4) for ^34^S and ^32^S), which govern seawater sulfate concentrations and its isotopic compositions:


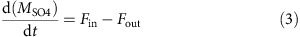


and


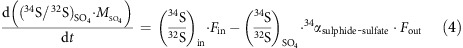


where ^34^*α*_sulfide-sulfate_ is the fractionation factor between sulfur buried as pyrite and sulfur buried as evaporite at the global scale, which is usually inferred from the average sedimentary Δ^34^S_SO4-pyr_ of equation (2), (ref. 10). Similar equations can be written for ^33^S and ^36^S isotope ratios. Because sulfur isotope fractionation factors ^33^*α* and ^36^*α* can be related to ^34^*α* by the ^33^*β* and ^36^*β* exponents, respectively (for example, ^33^*β*=ln(^33^*α*)/ln(^34^*α*), ref. 27), these equations can all be written as a function of ^34^*α*. Here for ^33^S:


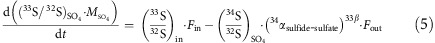


^33^*β* is typically close to 0.515 for abiotic processes and varies from 0.509 to 0.516 for microbial sulfate-reduction^28^,^29^. As illustrated by equations (4 and 5), the modelled Δ^33^S-δ^34^S trend of oceanic sulfate (blue line in Fig. 2) only depends on three parameters, namely *F*_in_/*F*_out_-ratio, ^33^*β* and ^34^*α*_sulfide-sulfate_ values. Most importantly, as opposed to previous approaches, in this model, ^34^*α*_sulfide-sulfate_ is a free parameter that we can explore to determine the best-fit scenario, that is, it is not deduced from the Δ^34^S_SO4-pyr_ values. The model does not depend on strong temporal constraints; therefore no *a priori* assumption was made on the initial sulfate residence time. Equally, no attempt was made to fit the isotope trend in Fig. 1, which would require a well-constrained deposition rate. However, we emphasize that the observed increase in δ^34^S_CAS_-values (and also Δ^33^S_CAS_, and δ^34^S_pyr_) through time is consistent with our model.

**Figure 3:**
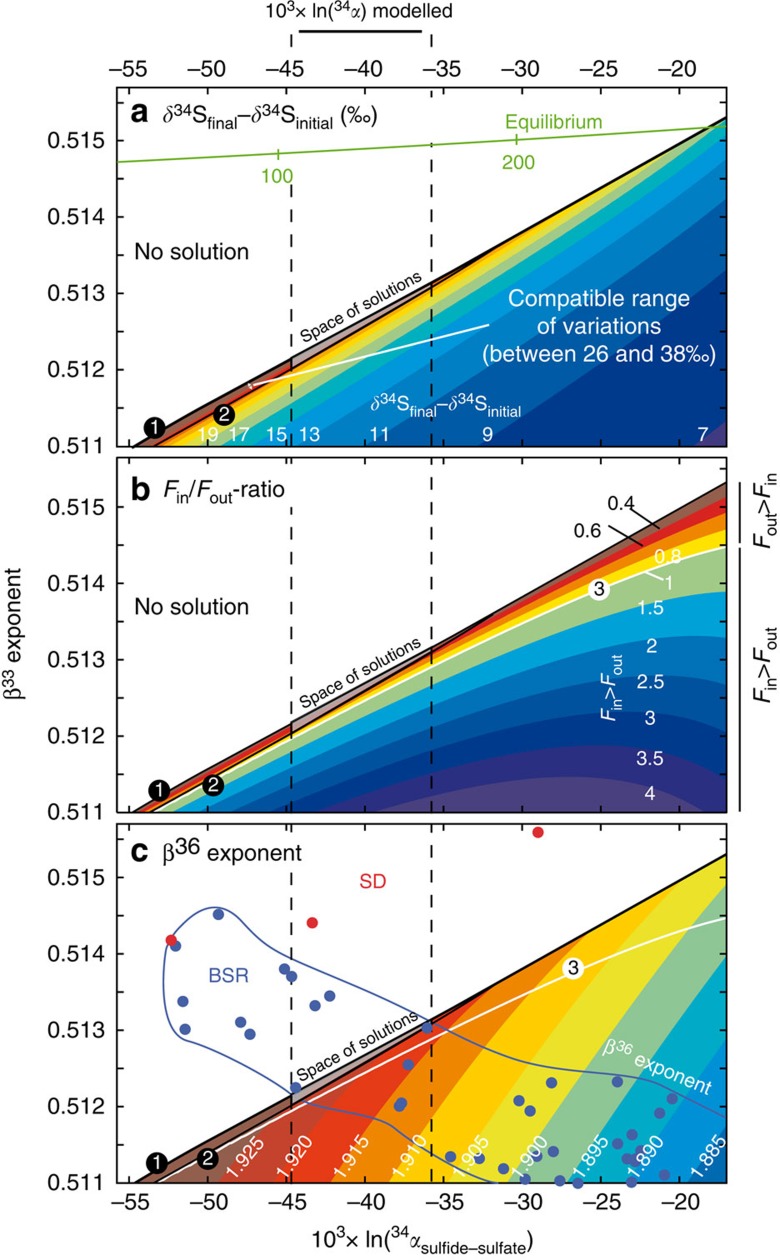
Model results in three 1,000ln(^34^*α*) versus ^33^*β*−exponents diagrams. The results show the combinations of ^33^*β*, ^34^*α* and *F*_in_/*F*_out_ that fit our data in 1,000ln(^34^α) versus ^33^*β*−exponents diagrams. (**a**) illustrates the δ^34^S_CAS_-range (that is, δ^34^S_CASfinal_−δ^34^S_CASinitial_) compatible with our data. The curve #2 delimitates the field below which the difference between δ^34^S_CASfinal_ and δ^34^S_CASinitial_ values (+38‰ and +12‰, respectively) is too low (that is, δ^34^S_CASfinal_−δ^34^S_CASinitial_<26‰) to account for the high δ^34^S_CAS_-values measured in our section. (**b**) illustrates the *F*_in_/*F*_out_-ratios that fit the observed δ^34^S_CAS_ versus Δ^33^S_CAS_ slope for each pair of ^33^*β*, ^34^*α*. The white curve #3 highlights the steady-state conditions, where *F*_in_=*F*_out_. (**c**) represents the combinations of ^33^*β* and ^34^*α* compatible with our data for different ^36^*β* values. The intercept between the modelled space of solution and results from BSR experiments allows restricting further the space of solution. The grey polygon represents the final solution space.

To better address the origin of the ^34^S-enriched signatures of oceanic sulfate, we investigated the variations in multiple sulfur isotope compositions for various combinations of *F*_in_/*F*_out_, ^33^*β* and ^34^*α*_sulfide-sulfate_ solving simultaneously equations (3, 4, 5). All parameters, namely ^33^*β*− and ^36^*β*− factors, *F*_in_/*F*_out_, ^34^*α*_sulfide-sulfate_ and S-isotope compositions of inputs were considered constant with time. Initial oceanic sulfate-sulfur isotope values were chosen from the data at the base of the sequence, which correspond to the onset of the observed excursion with δ^34^S_initial_∼+12‰, Δ^33^S_initial_=−0.016‰ and Δ^36^S_initial_=−0.1‰ (Fig. 2). The initial values may in fact have been lower and, in this respect, our approach is conservative. Note that they are consistent with the few other available Neoproterozoic sulfate multi-isotopic values obtained on Amadeus Basin (Australia), MacKenzie Fold Belt (Canada) and Oman (refs 14, 30). We explored the space of solutions for different combinations of *F*_in_/*F*_out_-ratio, ^33^*β*−factor and ^34^*α*_sulfide-sulfate_ by calculating the *F*_*i*n_/*F*_out_-ratio that best fits the δ^34^S−Δ^33^S slope defined by our data for a given set of ^34^*α* and ^33^*β* values. For that, we used 51 values of ^33^*β* (from 0.511 to 0.516) and 231 values of ^34^*α* (expressed as 1,000ln(^34^*α*) from −17‰ to −60‰). A total of 11,781 combinations of ^33^*β*, ^34^*α* and *F*_in_/*F*_out_ values compatible with the observed δ^34^S−Δ^33^S slope were produced (Fig. 3). We can restrict step by step the space of solution of our model using constraints available for δ^34^S_CASfinal_−δ^34^S_CASinitial_, which describes the range of δ^34^S_CAS_ variation during the excursion, and ^36^*β*, which is the exponent linking ^34^*α* and ^36^*α*-values [^36^*β*=ln(^36^*α*)/ln(^34^*α*)]. Each step is described below and the successive restrictions of the space of solution are shown in the 1,000ln(^34^*α*) versus ^33^*β*-exponent diagram of Fig. 3a–c.

Valid combinations of ^33^*β*, ^34^*α* and *F*_in_/*F*_out_ are restricted to the coloured, lower half-right of each panel of Fig. 3, which is delimited by curve #1. There are no viable solutions above curve #1 because the corresponding ^33^*β* and ^34^*α*-values would produce Δ^33^S too close to zero compared with our data (Fig. 2). The first constraint used here is the δ^34^S_CASfinal_−δ^34^S_CASinitial_ parameter. Combinations of ^33^*β*, and ^34^*α* compatible with our data for different δ^34^S_CASfinal_−δ^34^S_CASinitial_ values are shown in Fig. 3a. Curve #2 delimitates the field below which the difference between δ^34^S_CASfinal_ and δ^34^S_CASinitial_ values (+38‰ and +12‰, respectively) is too low (that is, δ^34^S_CASfinal_−δ^34^S_CASinitial_<26‰) to account for the high δ^34^S_CAS_-values measured in our section. Therefore, only results above curve #2 (that is, δ^34^S_CASfinal_−δ^34^S_CASinitial_>26‰) are valid. This further constrains the space of solution to between curves #1 and #2, with ^33^*β*-exponent <0.514 and 1,000ln(^34^*α*)<−30‰, as defined by the intercept between curves #1 and #2 (Fig. 3a).

Figure 3b represents the range of *F*_in_/*F*_out_-ratios compatible with our model. It illustrates the calculated *F*_in_/*F*_out_-ratios that fit the observed δ^34^S_CAS_ versus Δ^33^S_CAS_ slope for each pair of ^33^*β* and ^34^*α*. The white curve #3 highlights steady-state conditions, where *F*_in_=*F*_out_. This figure clearly shows that for the field defined by curves #1 and #2, *F*_in_/*F*_out_-ratios are always below unity (*F*_in_<*F*_out_), pointing to a decrease in oceanic sulfate concentration through time. This demonstrates that whatever the input parameters, the observed trends between δ^34^S_CAS_ versus Δ^33^S_CAS_ cannot be reproduced in a steady-state model (Supplementary Fig. 4). Taken together, Fig. 3a and Fig. 3b constrain the solution space to *F*_in_<*F*_out_ and ^33^*β*<0.514 (between curves #1 and #2). Another interesting outcome of the model is that *β*^33^-values are distinct from those expected for sulfate-reduction to hydrogen sulfide under thermodynamic equilibrium (green curve in Fig. 3a), a result consistent with previous studies^31^,^32^,^33^.

**Figure 4:**
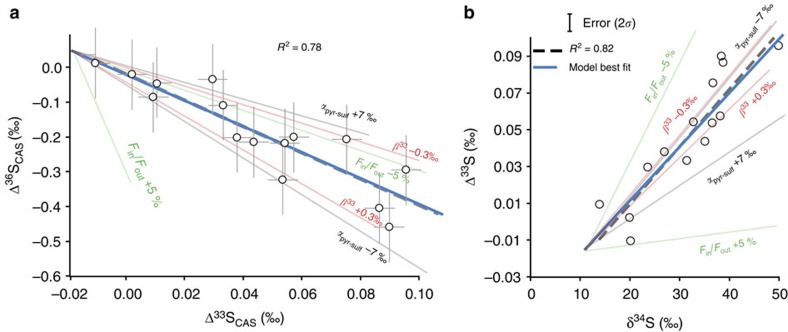
Sensitivity of the model to small variations of each parameter. (**a**) The Δ^36^S_CAS_ versus Δ^33^S_CAS_ diagram and (**b**) the Δ^33^S_CAS_ versus δ^34^S_CAS_ diagram. Changes in *F*_in_/*F*_out_ of ±5% (green line), in 1,000ln(^34^*α*) of ±7‰ (grey line) and in ^33^*β* of ±0.3‰ are tested. The blue line corresponds to the best-fit model and the dashed to the straight line passing through the data. This figure highlights the high sensitivity of the model to all considered parameters (that is, *F*_in_/*F*_out_, ^34^*α* and ^33^*β*). Based on duplicate and triplicate analyses, uncertainties of Δ^33^S and Δ^36^S values by the SF_6_ technique are estimated at 0.01‰ and 0.2‰ in 2*σ*, respectively.

**Figure 5:**
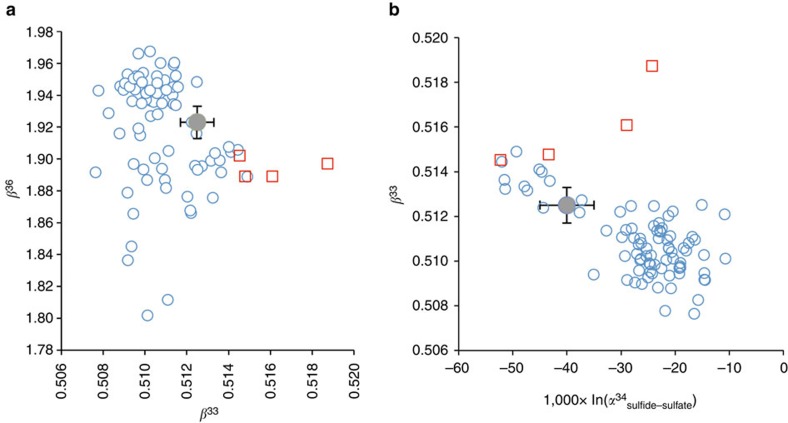
Multiple isotope results of our study compared with batch culture results. (**a**) ^33^*β* versus ^36^*β* diagram. (**b**) ^33^*β* versus 1,000ln(^34^*α*_sulfide-sulfate_) diagram. Blue circles are data from BSR culture experiments, (refs 28, 29, 34, 35, 36, 37). Red squares are data from SD culture experiments, ref. 29. The grey circle represents the results from our model (with the associated error bars, corresponding to the intersect between the solution space-Fig. 3a,b, and the field defined by culture experiments-Fig. 3c).

The space of solution can be further restricted taking into account the fact that ^36^*β* must also fit the observed Δ^33^S versus Δ^36^S correlation (Fig. 4a). Figure 3c represents the combinations of ^33^*β* and ^34^*α* compatible with our data for different ^36^*β*-values. Our space of solutions (between curves #1 and #2) is compared with ^34^*α*_sulfide-sulfate_ data and their respective ^33^*β* and ^36^*β* exponents estimated from batch culture experiments for the two main sulfur metabolisms, namely SD and BSR (refs 28, 29). Our space of solutions intersects only the field delimited by the BSR data set while SD data (red dots in Fig. 3c) plot outside. This allows us to assume that BSR is the main mechanism leading to sulfate drawdown and to further restrict our space of solutions to its intersection with the BSR cultures data set. It is worth noting, however, that available data from culture experiments are still limited and present significant variability. In Fig. 5, all available ^34^α_sulfide-sulfate_, ^33^*β* and ^36^*β*-values data for culture experiments^28^,^29^,^34^,^35^,^36^,^37^ are plotted together with our modelled values (full grey circle). Although available data most clearly relate to BSR, one cannot rule out that part of the signal may reflect a mixed signature between SD and BSR organisms (Fig. 5).

The final space of solutions in Fig. 3 is represented by a grey polygon which corresponds to the following combination of values: ^34^*α*_sulfide-sulfate_∼0.960±0.005, ^33^*β*∼0.5125±0.0005 and *F*_in_/*F*_out_=0.30±0.25. The high sensitivity of the model allow to have precise values for the best-fit scenario, indeed slight modifications of each modelled parameter (^34^*α*_sulfide-sulfate_, ^33^*β* and *F*_in_/*F*_out_) shows significant effects on the δ^34^S_CAS_ versus Δ^33^S_CAS_ slope (Fig. 4b).

This multi-isotopic approach allows us, using the best-fit values deduced above for ^33^*β*, ^34^*α* and *F*_in_/*F*_out_ in equations (4 and 5), to quantify the contraction of the sulfate reservoir responsible for the observed increase in both δ^34^S_CAS_ and Δ^33^S_CAS_ (blue line in Fig. 2). For the strong increase in δ^34^S-value from +12‰ at the base of the section to +38‰ at the top, the model indicates that water column sulfate concentrations decreased dramatically by nearly 50%. In other words, at the end of deposition of the Guia Formation, sulfate concentration would be only half its initial value (Fig. 1). A single extreme δ^34^S_CAS_ value of +50‰ is observed in a typical event bed at the top of the section^17^, characterized by hummocky cross stratification. Given its sedimentary characteristics, it is unclear whether this single extreme value should be considered; if representative, it would indicate a 60% decrease compared with the sulfate concentration observed at the base of the Araras composite section. The high δ^34^S values reported in post-Marinoan glacial deposits from Namibia^7^ and North China^26^ can also be accounted for by a drawdown of oceanic sulfate in a dynamic non steady-state sulfur cycle, without invoking the extreme modifications in both *f*_pyr_ and δ^34^S_in_ needed in a steady-state model approach.

The model also provides fundamental constraints on the lower limit of marine sulfate concentration during the Neoproterozoic. For sulfate concentrations below 1 mM, the sulfur isotope fractionation associated with BSR decreases, reaching 0‰ below 200 μM (ref. 38) or lower^39^. The results showing ^34^*α*_sulfide-sulfate_ close to 0.960 (Δ^34^S=−40‰) without significant changes throughout the sequence, suggests that sulfate concentrations remained well above 200 μM even by the end of the sulfate reservoir drawdown. The fractionation factor would otherwise have decreased significantly^38^. This constrains the lower limit of marine sulfate concentration in the immediate aftermath of the glaciation to well above 400 μM. These estimates are consistent with the work of Kah *et al*.^13^, who constrained the upper limits of Neoproterozoic marine sulfate concentrations to between 7 and 10 mM using a completely independent approach.

The post-glacial marine environment recorded in the Snowball aftermath deposits is thought to be characterized by an enhanced delivery of phosphate^40^ and bioavailable iron^41^. The resulting planktonic bloom suggested by Elie *et al*.^42^ accompanying the Snowball deglaciation may have increased the organic matter flux to the sediment exhausting its dissolved O_2_ content and enhancing anaerobic respiration of organic matter. We thus suggest here that post-glacial conditions were adequate for anaerobic sulfate-reduction metabolism triggering significant sulfate removal from the water column. If widespread, such a sulfate drawdown by BSR and pyrite burial would have important consequences for other biogeochemical cycles including the global oxygen budget during the end of the Neoproterozoic Era^2^,^43^. Pyrite burial plays a key role in the long-term sources of atmospheric O_2_ and its transient increase may have contributed to the accumulation of O_2_ in the oceans and atmosphere^44^,^45^. While burial of organic matter is generally considered to be the most important driver of O_2_ production on geological timescales (present-day value 10 × 10^12^ mol O^2^ per year), refs 44, 45, today pyrite burial contribution to this long-term redox balance is of the same order of magnitude^44^,^45^.

We therefore estimated a minimum range of O_2_ fluxes produced by the post-glacial intense BSR activity and pyrite burial implied by our model. Because the flux of sulfate delivery to the ocean (*F*_in_) is unknown for this period, we only calculate the net O_2_ flux due to a 50% decrease of the ocean sulfate concentration without continuous sulfate inputs to the ocean. In that sense the fluxes in the following calculation has to be taken as minimum values. From a global perspective and assuming the modern ocean volume^46^, the combined results of our study and others^13^,^46^,^47^,^48^ constrain the total marine sulfate reservoir of the late Neoproterozoic to between 5 × 10^17^ mol and 13 × 10^18^ mol (SO_4_^2−^ concentrations of 0.4–10 mM, respectively). Considering the stoichiometry of BSR coupled to pyrite precipitation, where reduction of 1 mol SO_4_^2−^ and burial as pyrite equates the release of 2 mol O_2_, ref. 49, a 50% decrease in the late Neoproterozoic sulfate reservoir by BSR coupled to pyrite burial is equivalent to the net total production of 0.5–13 × 10^18^ mol of O_2_.

**Figure 6:**
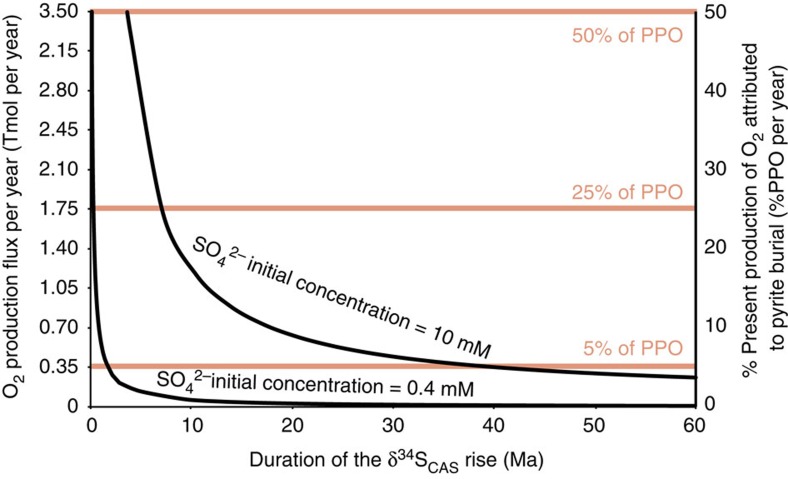
O_2_ fluxes versus duration of the sulfur isotopic excursion. O_2_ fluxes produced by a 50% distillation of the oceanic sulfate reservoir by sulfate-reduction and pyrite burial as a function of the duration of the sulfur isotopic excursion. For the bottom curve the starting concentration of sulfate was of 0.4 mM, for the top one it was of 10 mM. The *y* axis to the right corresponds to the proportion of O_2_ produced compared with the present production of O_2_ attributed to pyrite burial per year (PPO/yr), estimated at 7 Tmol per year by Holland *et al*.^44^

To express this number as a flux of O_2_, one needs tight temporal constraints that are lacking for the Araras Group (see above). Figure 6 presents the flux of O_2_ produced from pyrite buried per year as a function of the duration of the δ^34^S_CAS_ increase episode. Using the conservative deposition time of 60 Myr, the decrease in sulfate concentration observed would lead to an O_2_ production comprised between 0.1 and 1.2 × 10^12^ mol per year (Fig. 6). These values correspond respectively to 0.1 and 3.2% of the modern production of O_2_ attributed to pyrite burial per year (Fig. 6)^49^,^50^. If the increase in δ^34^S-values has occurred over a shorter interval of time, which is supported by the fact that the observed increase in δ^34^S-values occurs within the first 175 m of the section, the O_2_ flux is then higher. For a depositional time period of 10 Myr, the O_2_ production flux would rise to between 1.5 and 20% of the present O_2_ production by BSR activity (Fig. 6). We conclude, therefore, that in the aftermath of the Marinoan glaciation enhanced BSR and pyrite burial represents a viable mechanism contributing to the Neoproterozoic oxygenation event of the ocean-atmosphere system.

## Methods

### Sample preparation

Five to 100 g of carbonate samples (with carbonate contents typical >70 wt% of the total rock) were powdered, leached of soluble sulfates in a 5% NaCl solution, followed by four rinses in deionized (DI) water. This step was repeated three times, then the powder was dissolved in 4 N HCl (12 h). The acidified samples were filtered, on a 0.45-μm nitrocellulose paper and an excess of 250 g l^−1^ of BaCl_2_ was added to the filtrate to precipitate BaSO_4_.

### Multiple sulfur analyses

The samples were prepared and analysed for their multiple sulfur isotope compositions at the Stable Isotope Laboratory of the Institut de Physique du Globe de Paris.The barium sulfate was subsequently reacted with Thode reagent^51^ in a helium atmosphere extraction line. The released H_2_S was converted to silver sulfide (Ag_2_S) by reaction with a silver nitrate solution and silver sulfide was fluorinated to produce SF_6_. The δ^34^S values are presented in the standard delta notation against V-CDT with an analytical reproducibility of≤0.1‰. We report these values against an assumed Δ^33^S and Δ^36^S for Vienna Cañon Diablo troilite (V-CDT) that yields δ^34^S, Δ^33^S and Δ^36^S values for IAEA S-2 (*n*=23) of 5.224‰, 0.030 and −0.203‰, respectively. Based on duplicate and triplicate analyses, uncertainties of Δ^33^S and Δ^36^S values by the SF_6_ technique are estimated at 0.01 and 0.2‰ in 2*σ*, respectively.

### Model and concepts associated with non-zero Δ^33^S and Δ^36^S

The plot of ^33^S/^32^S and ^34^S/^32^S fractionations displays a slight curvature expressed by:


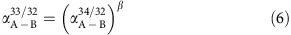


The β-exponent is not arbitrary and can be deduced from the high-temperature approximation of the reduced partition function, from the mass (in atomic mass unit) of the considered isotopes, for example, ref. 52. Thus, for ^33^S/^32^S and ^34^S/^32^S fractionation ^33^β corresponds to:


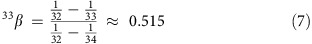


Exception made of a few molecules showing little relevance in the present study^27^,^53^, and discussion in ref. 54, equilibrium isotope fractionation at any temperature show β-values close to the high-temperature approximation^27^,^53^. Using δ notation one can write:


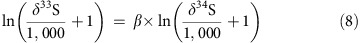


A mixing between two isotopically different pools (A and B) will fall along a mixing line (equations (9); ref. 55) that deviates from the theoretical equilibrium curve:


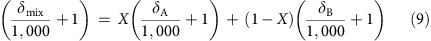


where X denotes for the fraction of A. In other words, two sulfur reservoirs at isotope equilibrium will lie on the same fractionation line, with the same Δ^33^S. In contrast mixing will be expressed along a secondary fractionation line with negative Δ^33^S. The resulting Δ^33^S-anomaly is maximum for 50% mixing being approximately −0.05‰ for two pools differing by 40‰ in δ^34^S.

Given that the mixing of two pools leads to negative Δ^33^S, the formation of two sulfur pools at isotope equilibrium will move them along a secondary fractionation line above that of the starting composition. These effects can be enhanced by Rayleigh distillation (that is, open system fractionation) and can be demonstrated starting from the well-known equation^56^:


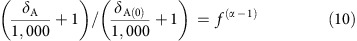


With δ_A_ the isotopic composition of the residual component of A and and δ_A(0)_ being the starting isotope composition, *f* is the residual sulfate concentration in our case, and *α* is the isotopic fractionation corresponding to the given system studied (bacterial sulfato-reduction in our study).

### Data availability

All results that support the findings of this study are available in Supplementary Table 1.

## Additional information

**How to cite this article:** Sansjofre, P. *et al*. Multiple sulfur isotope evidence for massive oceanic sulfate depletion in the aftermath of Snowball Earth. *Nat. Commun.* 7:12192 doi: 10.1038/ncomms12192 (2016).

## Supplementary Material

Supplementary Information Supplementary Figures 1-4, Supplementary Table 1 and Supplementary References.
