# Supplementary material for: Multiple sulfur isotope evidence for massive oceanic sulfate depletion in the aftermath of Snowball Earth
Source: Nat Commun. 2016 Jul 22;7:12192. doi: 10.1038/ncomms12192 (PMC4961837; doi:10.1038/ncomms12192)
Supplement: Supplementary Information — Supplementary Figures 1-4, Supplementary Table 1 and Supplementary References. [file ncomms12192-s1.pdf]

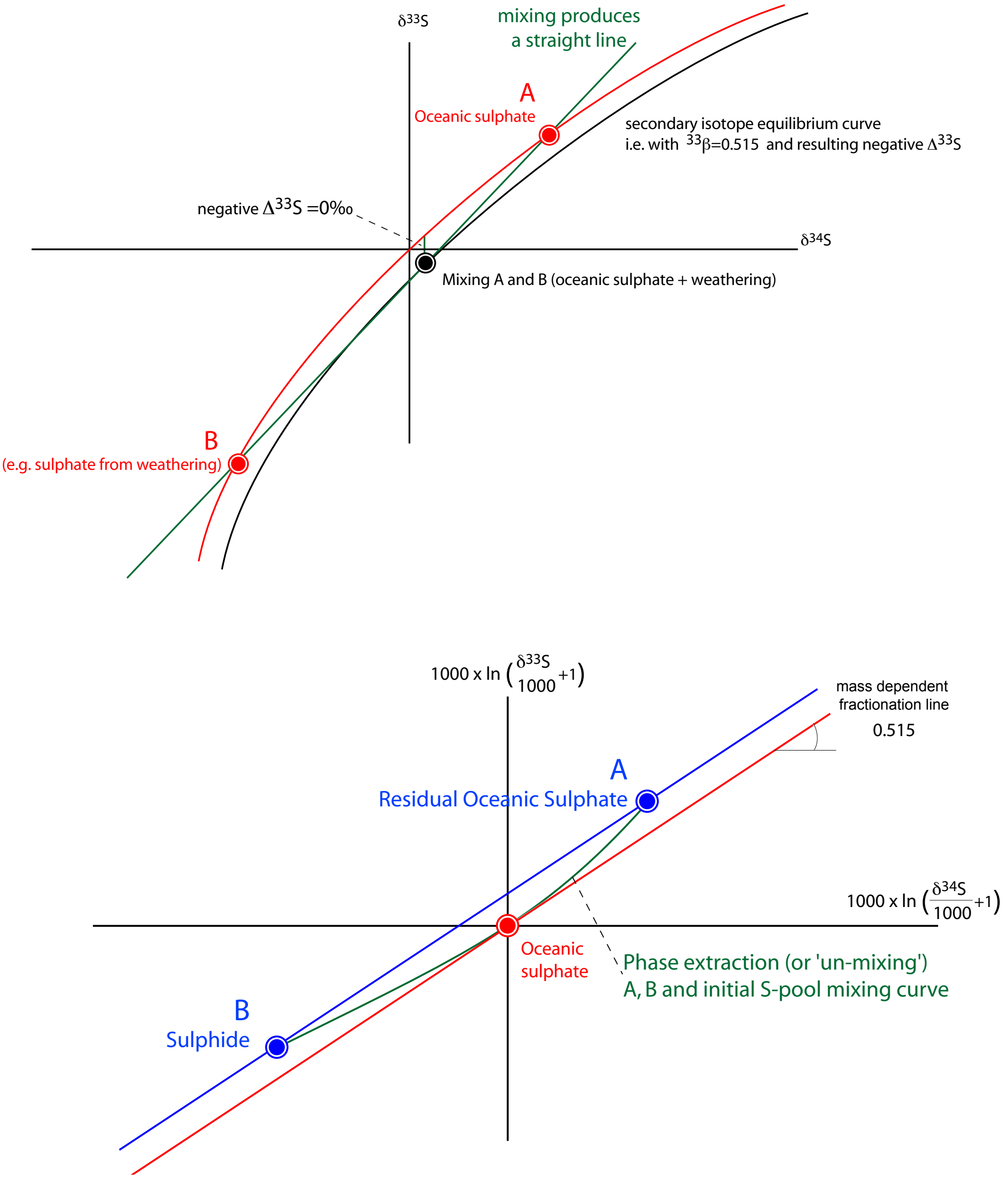


**Supplementary Figure 1:** Two representations of the relationship between $\delta^{33}S$, $\delta^{34}S$ and non-zero $\Delta^{33}S$-values resulting for a mixing of two pools with different sulphur isotopic composition, yet with $\Delta^{33}S$ = 0‰. Green line is derived from equation [9]. Figure not to scale.

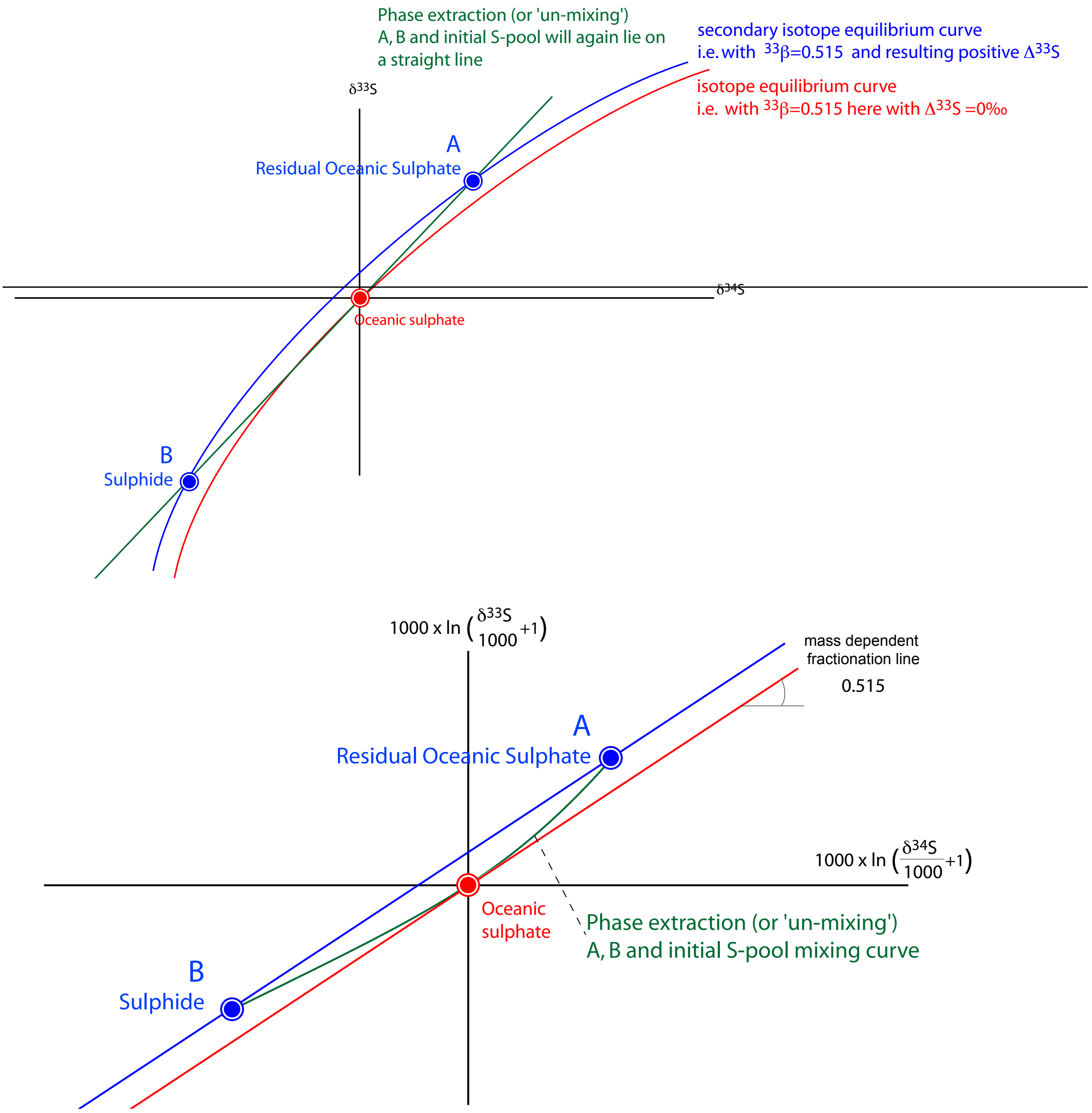


**Supplementary Figure 2**: Two representations of the relationship between $\delta^{33}$S, $\delta^{34}$S and non-zero $\Delta^{33}$S-values resulting for phase extraction (or un-mixing) of two pools A and B at isotope equilibrium from a reservoir with $\Delta^{33}S = 0‰$. Mass conservation effects lead both A and B to have slightly positive $\Delta^{33}$S. Green line is derived from equation [9]. Figure not to scale.

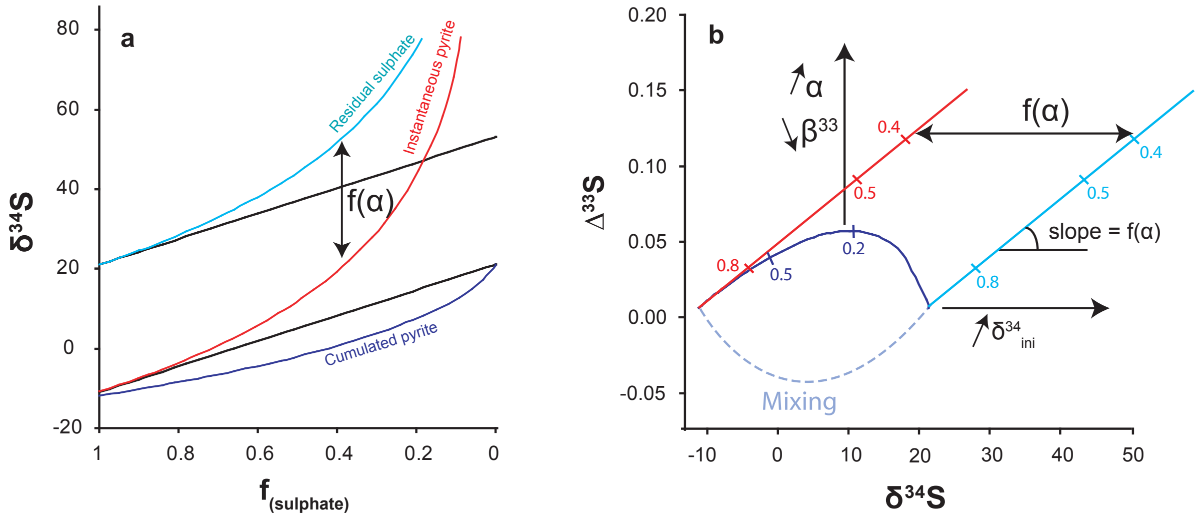


**Supplementary Figure 3**: Schematic isotopic evolution of the residual sulphate reservoir, instantaneous and cumulative pyrite during a Rayleigh (i.e. open-system) distillation process. **a)** $\delta^{34}S$ vs $f_{(sulphate)}$ calculated from equation [10]. **b)** $\Delta^{33}S$ vs $\delta^{34}S$ calculated from equations [8 and 10] for a $^{34}\alpha_{pyr\text{-}sulph}$ of 0.970 and an initial $\delta^{34}S$ of 21 ‰. All calculations assume $^{33}\beta$-values corresponding to equilibrium (i.e. 0.515).

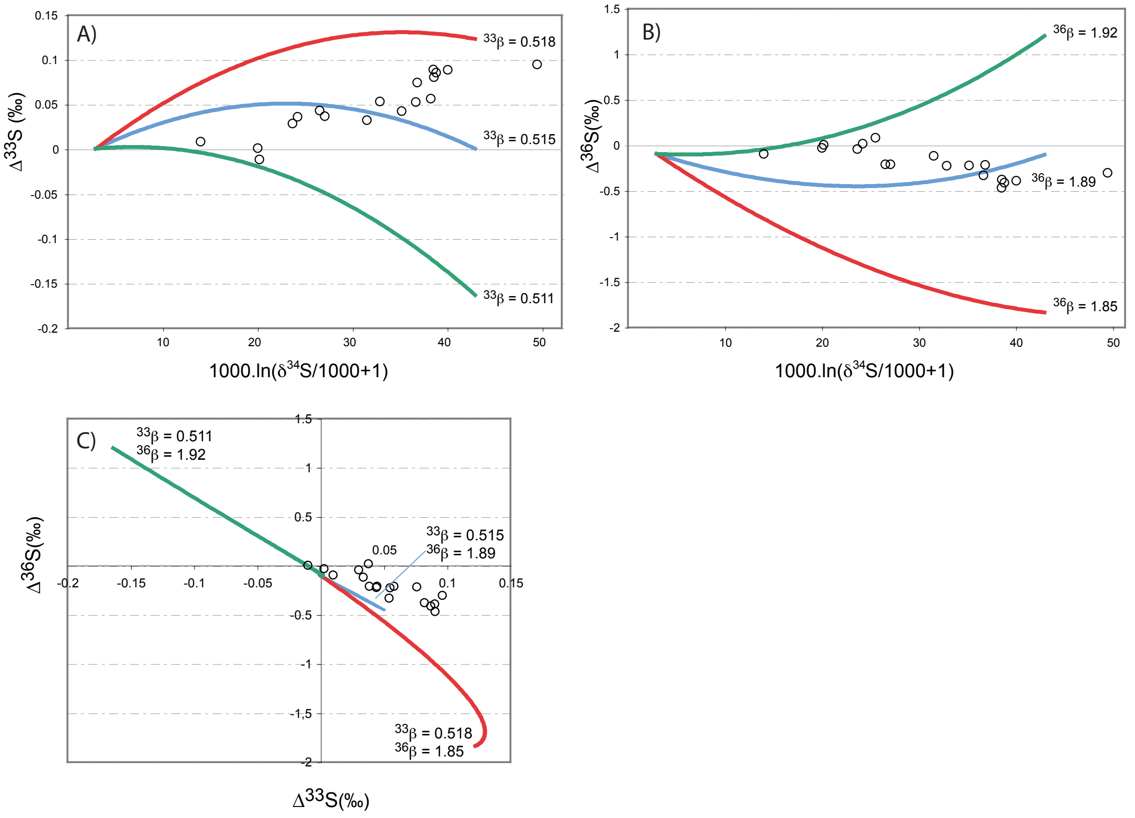


**Supplementary Figure 4**: A), B) and C) $\delta'^{34}S$ and $\Delta^{33}S$ and $\Delta^{36}S$ relationships predicted for sulphur isotope compositions of oceanic sulphate in a steady state assumption determined for a series of $^{33}\beta$-values. Open circles are data obtained in this study and given in the Supplementary Table 1.

**<u>Supplementary Table 1</u>**: **Sample** is the name of the sample. CRS are samples from Carmelo quarry. TeS from Terconi quarry and CA and CAS from Camil quarry. **$\delta^{34}S$. $\Delta^{33}S$ and $\Delta^{36}S$** are expressed in ‰ vs CTD. **$FeS_2$** is the content of pyrite in %. **CAS** is the content of Carbonate Associated Sulphate in ppm. Nd indicates that the CAS content was not determined. Johnston et al. 2005 indicates these data have been compiled from Johnston et al. 2005. Errors are given in the method section.

| Sample | $\delta^{34}S_{pyr}$ | $\Delta^{33}_{pyr}$ | $\Delta^{36}_{pyr}$ | $FeS_2$ |
|---|---|---|---|---|
| CRS 0 | -8,97 | 0,10 | -0,10 | 0,01 |
| CRS 3 | -9,87 | -0,02 | -0,20 | 0,07 |
| CRS 5 | -5,04 | -0,06 | 0,17 | 0,09 |
| CRS 11 | -3,84 | 0,04 | -0,34 | 0,03 |
| CRS 13 | -3,22 | 0,03 | -0,73 | 0,75 |
| CRS 16 | -5,37 | 0,07 | -0,58 | 3,06 |
| CRS 19 | 12,21 | 0,09 | -0,77 | 0,15 |
| CRS 21 | 26,21 | 0,06 | -0,37 | 0,05 |
| CRS 23 | 1,65 | 0,06 | -0,74 | 0,54 |
| CRS 30 | -3,93 | 0,16 | -1,39 | 3,01 |
| CRS 31 | 0,32 | 0,11 | -0,88 | 0,84 |
| CRS 32 | 8,94 | 0,08 | -0,83 | 0,43 |
| CRS 35 | 16,97 | 0,07 | -0,58 | 0,18 |
| CA -0.63 | 8,88 | 0,06 | -0,72 | 0,30 |
| CA -6.65 | 3,74 | 0,08 | -0,71 | 1,03 |
| CA -20.6 | 4,36 | 0,04 | -0,49 | 0,44 |
| CAS -34 | 0,39 | 0,05 | -0,55 | 0,53 |
| CAS -40 | 2,91 | 0,03 | -0,36 | 0,53 |
| CAS -60 | 8,28 | 0,04 | -0,50 | 0,32 |
| CAS -80 | 7,07 | -0,01 | -0,30 | 0,25 |
| CAS- 96 | 6,13 | 0,02 | -0,40 | 0,12 |
| CAS -106 | 11,61 | 0,06 | -0,51 | 0,64 |
| CAS -114 | 7,65 | 0,06 | -0,50 | 0,17 |
| TeS 22 | 17,23 | 0,00 | -0,13 | 0,33 |
| TeS 24 | 25,67 | 0,04 | -0,31 | 0,76 |
| TeS 26 | 24,73 | 0,02 | -0,17 | 0,40 |
| TeS 28 | 24,78 | 0,06 | -0,39 | 0,60 |
| TeS 30 | 25,38 | 0,01 | -0,08 | 0,81 |
| TeS 32 | 15,83 | 0,04 | -0,52 | 0,69 |
| TeS 34 | 21,71 | 0,04 | -0,32 | 0,64 |
| TeS 36 | 13,57 | 0,05 | -0,60 | 0,71 |
| TeS 38 | 17,90 | 0,01 | -0,28 | 0,60 |
| TeS 46 | 28,85 | -0,03 | 0,32 | 0,23 |
| TeS 48 | 18,66 | -0,01 | -0,15 | 0,18 |
| TeS 50 | 25,90 | -0,02 | 0,19 | 0,13 |

| Sample | $\delta^{34}S_{CAS}$ | $\Delta^{33}_{CAS}$ | $\Delta^{36}_{CAS}$ | CAS |
|---|---|---|---|---|
| CRS 1 | 23,88 | 0,03 | -0,03 | 98 |
| CRS 2 | 16,52 | 0,03 | -0,43 | Barite |
| CRS 8 | 33,35 | 0,03 | -0,43 | 13 |
| CRS 13 | 31,95 | 0,03 | -0,11 | 40 |
| CRS 16 | 27,37 | 0,04 | -0,20 | 74 |
| CRS 19 | 44,00 | Nd | 0,09 | 69 |
| CRS 21 | 50,64 | 0,10 | -0,30 | 25 |
| CRS 23 | 37,44 | 0,08 | -0,21 | 16 |
| CRS 25 | 35,74 | 0,04 | -0,22 | 24 |
| CRS 28 | 38,94 | 0,06 | -0,20 | 34 |
| CRS 32 | 39,20 | 0,09 | -0,46 | 68 |
| CRS 35 | 37,27 | 0,05 | -0,32 | 23 |
| CAS -60 | 39,49 | 0,09 | -0,40 | 106 |
| TeS 9 | 14,02 | 0,01 | -0,09 | Nd |
| TeS 32 | 20,15 | 0,00 | -0,02 | 69 |
| TeS 40 | 20,33 | -0,01 | 0,01 | 25 |
| Johnston et al 2005[ref.1] | 17,03 | -0,02 | -0,02 | Nd |
| Johnston et al 2005[ref.1] | 16,42 | -0,01 | -0,10 | Nd |
| Johnston et al 2005[ref.1] | 17,42 | -0,01 | -0,02 | Nd |
| Johnston et al 2005[ref.1] | 20,97 | -0,01 | -0,18 | Nd |
| Johnston et al 2005[ref.1] | 23,10 | 0,00 | -0,16 | Nd |
| Johnston et al 2005[ref.1] | 19,94 | 0,01 | -0,12 | Nd |
| Johnston et al 2005[ref.1] | 21,39 | 0,01 | -0,05 | Nd |
| Johnston et al 2005[ref.1] | 19,15 | 0,02 | -0,17 | Nd |

**Supplementary Reference:**

1. Johnston, D. T., Wing, B. A., Farquhar, J., Kaufman, A. J., Strauss, H., Lyons, T. W. & Canfield, D. E. Active microbial sulfur disproportionation in the Mesoproterozoic. *Science* **310**, 1477-1479 (2005).
